# Arsenic speciation in rice bran: Agronomic practices, postharvest fermentation, and human health risk assessment across the lifespan^☆^

**DOI:** 10.1016/j.envpol.2021.117962

**Published:** 2021-12-01

**Authors:** Annika M. Weber, Bridget A. Baxter, Anna McClung, Molly M. Lamb, Sylvia Becker-Dreps, Samuel Vilchez, Ousmane Koita, Frank Wieringa, Elizabeth P. Ryan

**Affiliations:** a Department of Food Science and Human Nutrition, Colorado State University, Fort Collins, CO 80523, USA; b Department of Environmental and Radiological Health Sciences, Colorado State University, Fort Collins, CO 80523, USA; c USDA-Agricultural Research Service, Dale Bumpers National Rice Research Center, Stuttgart, AR 72160, USA; d Department of Epidemiology and Center for Global Health, University of Colorado School of Public Health, Aurora, CO 80045, USA; e Departments of Family Medicine and Epidemiology, University of North Carolina at Chapel Hill, Chapel Hill, NC, 27599-7595, USA; f Center of Infectious Diseases, Department of Microbiology and Parasitology, Faculty of Medical Sciences, National Autonomous University of Nicaragua, León (UNAN-León), León, Nicaragua; g Laboratoire de Biologie Moléculaire Appliquée, Campus de Badalabougou, Université des Sciences, des Techniques et des Technologies de Bamako, BP: 1805, Bamako, Mali; h Alimentation, Nutrition, Santé (E6), UMR95 QualiSud, University of Montpellier, Avignon Université, CIRAD, Institut Agro, Institut de Recherche pour le Développement (IRD), Université de La Reunion, Montpellier, France

**Keywords:** Arsenic, Inorganic arsenic, Speciation, Rice bran, Food safety, Health risk assessment

## Abstract

Arsenic (As) exposure is a global public health concern affecting millions worldwide and stems from drinking water and foods containing As. Here, we assessed how agronomic practices and postharvest fermentation techniques influence As concentrations in rice bran, and calculated health risks from consumption. A global suite of 53 rice brans were tested for total As and speciation. Targeted quantification of inorganic As (iAs) concentrations in rice bran were used to calculate Target Hazard Quotient (THQ) and Lifetime Cancer Risk (LCR) across the lifespan. Mean iAs was highest in Thailand rice bran samples (0.619 mg kg^−1^) and lowest in Guatemala (0.017 mg kg^−1^) rice bran samples. When comparing monosodium-methanearsonate (MSMA) treated and the Native-soil counterpart under the irrigation technique Alternate Wetting and Drying (AWD) management, the MSMA treatment had significantly higher total As (*p* = 0.022), and iAs (*p* = 0.016). No significant differences in As concentrations were found between conventional and organic production, nor between fermented and non-fermented rice bran. Health risk assessment calculations for the highest iAs-rice bran dosage scenario for adults, children and infants exceeded THQ and LCR thresholds, and LCR was above threshold for median iAs-rice bran. This environmental exposure investigation into rice bran provides novel information with food safety guidance for an emerging global ingredient.

## Highlights

• Rice bran arsenic speciation was quantified across a globally diverse sample set.
• Agronomic methods such as irrigation influenced arsenic concentrations in rice bran.
• Postharvest rice bran fermentation minimally impacted arsenic concentrations.
• Human health risk assessments suggest some rice bran varieties merit dosage regulation.

## Graphical abstract

**Figure:**
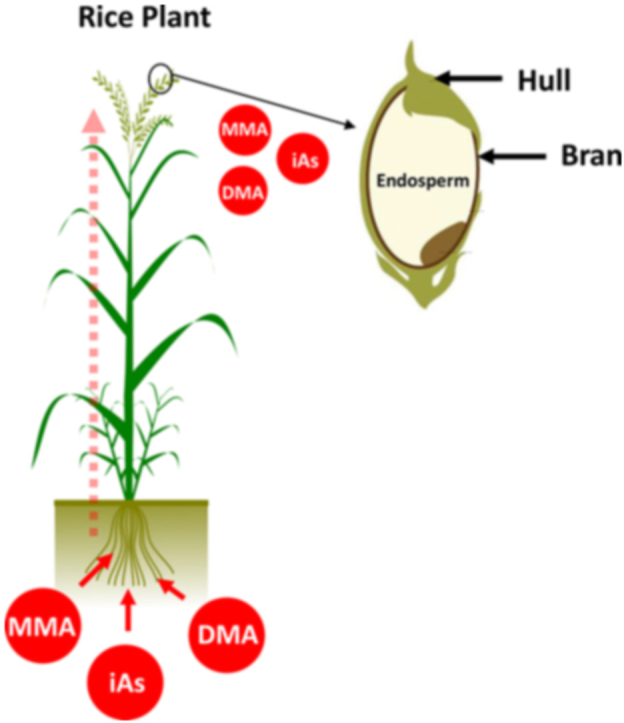

## Abbreviations

As: Arsenic
As(III): Arsenite
As(V): Arsenate
AWD: Alternate Wetting and Drying
DMA: Dimethylarsinic acid
iAs: Inorganic Arsenic
LCR: Lifetime Cancer Risk
MMA: Monomethylarsonic acid
MSMA: Monosodium-methanearsonate
THQ: Target Hazard Quotient

## 1 Introduction

Rice bran is an emerging “superfood” rich in fatty acids, phytochemicals, B and E vitamins, and soluble and insoluble prebiotic fibers (Ghasemzadeh et al., 2018; Ryan, 2011). With its antioxidant and anti-microbial properties (Goodyear et al., 2015), rice bran may have protective effects against colon cancer (Henderson et al., 2012b) and enteric infections (Kumar et al., 2012; Lei et al., 2016), improve gut mucosal immune protection (Henderson et al., 2012a), and support linear growth in infants (Zambrana et al., 2019). Alongside these health benefits, concern exists over dietary exposures of arsenic (As) from rice bran. Studies have shown that total As concentrations were higher in brown rice than in white, polished rice from the same paddy field, and that reduction in As concentration was related to removal of the bran layer (Naito et al., 2015; Sun et al., 2008). Elemental mapping of As in the rice grain using μ-XRF and μ-XANES demonstrated that As can accumulate in the husk and bran layer (Meharg et al., 2008; Wu et al., 2019). Arsenic toxicity is largely species dependent, with inorganic species As(III) and As(V) considered to be the most toxic forms, and organic species exhibiting mild or no toxicity in humans (Akter et al., 2005; Chiocchetti et al., 2019). Inorganic As (iAs) and its methylated derivatives dimethylarsinic acid (DMA) and monomethylarsonic acid (MMA) are the dominant species found in crops, including rice grain (Kim et al., 2013; Torres-Escribano et al., 2008; Zavala and Duxbury, 2008).

Arsenic is naturally occurring in soil, with concentrations and availability for plant uptake varying widely based on geographic location, soil properties, soil microbial populations, and complex plant-soil interactions (Punshon et al., 2017). Regions of the world such as Bangladesh have shown high levels of geogenic As resulting in high concentrations in water and food (Islam et al., 2018; Meharg et al., 2009). Anthropomorphic sources can also impact soil contents of As. Arsenic based pesticides have limited use today, but previously were widely used to control insect pests and the herbicide monosodium methanearsonate (MSMA) has been used to control weeds resulting in possibly high residual levels of As in the soil and water (Gilmour and Wells, 1980; Missimer et al., 2018). Irrigation technique has also been recognized to influence soil As availability, whereby traditional flooded paddy fields create an anaerobic environment, conducive for iAs uptake by rice plants (Williams et al., 2007). An irrigation approach that has been implemented that mitigates As accumulation in rice is the alternate wetting and drying (AWD) practice. This introduces periods of intermittent drying throughout the rice growing season, creating aerobic intervals, and thereby decreasing iAs uptake from soil by plants (Carrijo, 2018; Li et al., 2019).

Unlike conventional rice cultural management which utilizes herbicides to augment weed control, certified organic rice production does not allow their use and thus, maintaining season-long flooded paddies is a critical weed control method. Recent As rice accumulation studies have demonstrated that organic rice can have higher levels of total and iAs as compared to conventionally produced samples (Ma et al., 2014; Menon et al., 2020; Segura et al., 2016). This may be related to the extended duration that fields are flooded and anerobic, as well as cultural practices that increase organic matter and soil health (e.g. incorporation of straw or animal manure) (Hossain et al., 2021; Ma et al., 2014; Norton et al., 2013). Market survey results of As in rice are not consistent (Poletti et al., 2014) and may reflect the diverse spectrum of organic production methods, environments, and post-harvest processing treatments. Additionally, post-harvest processing, including fermentation is a potential technique in reducing heavy metal exposure in food (Massoud et al., 2019). Arsenic bioremediation via application of microbial fermentation is still being analyzed for how As concentrations may be altered in fermented rice products (Signes-Pastor et al., 2009).

While studies have analyzed As concentrations in rice grains, little research emphasis exists for rice bran that is an emerging superfood for human health. Thus, it is imperative that As concentrations and speciation for rice bran be quantified. Here, we aimed to provide an in-depth analysis of pre- and postharvest techniques including geographic origin, agronomic cultural practices, as well as probiotic fermentation, to assess their influence on As concentration and speciation in a global suite of rice brans. Human health risk assessment calculations were performed to identify safe consumption amounts that have not been previously considered.

## 2 Methods

### 2.1 Sample sourcing and preparation

A set of 53 rice bran samples were obtained from rice research stations, local growers, rice millers, and rice bran industry suppliers during 2011–2020 which were produced from rice grown in 10 countries, some having multiple growing environments. The details of the geographic origin, variety name, cultural management treatment, and bran color are listed in Table S1. The global suite of rice sources and rice brans (USA n = 20, Cambodia n = 6, India n = 3, Egypt n = 1, Guatemala n = 2, Mali n = 1, Madagascar n = 2, Nepal n = 1, Nicaragua n = 1, Thailand n = 1) were procured for use in arsenic quantification and human health risk assessment. Rice bran came from growing practices typical for the country or conventional growing (n = 28), organic cultural practices in the US and in Thailand (n = 6), combinations of irrigation techniques and soil treatment with or without MSMA applied to the soil (AWD/Native soil n = 2, AWD/MSMA n = 2, Flooded/Native soil n = 2, Flooded/MSMA n = 2), and targeted probiotic fermentations (n = 11). A total of 53 rice bran samples were used in multiple comparisons of agronomic conditions and for use in calculations of health risk assessments.

### 2.2 Global rice varieties

Rice bran samples from Guatemala, Madagascar, Nicaragua, and Cambodia were collected from local mills and heat stabilized prior to sending for analysis. The Cambodian rice bran samples were collected from 2 different mills (indicated as I and II in Table S1). The rice bran sample from New Delhi, India was collected from a local mill and was not heat stabilized. The Egyptian rice bran sample was provided by Spica BV, Netherlands and was heat stabilized prior to shipment. The varieties Chennula and Njavara, and samples from Nepal, and Mali were collected as rough rice, and were milled and heat stabilized at Colorado State University, according to details previously described (Zarei et al., 2018). Rice bran of the variety Urmatt from Thailand and the RBT-300 market samples I and II, from Calrose medium grain produced in California (Zarei et al., 2018), were provided by Rice Bran Technologies, Sacramento, CA (Velasquez-Munoz et al., 2019). These samples were heat stabilized prior to shipment. All rice bran samples were stored at −20 °C until processed for analysis of As concentrations.

### 2.3 Wells and IAC600 rice bran: irrigation and soil treatment

Two varieties, Wells and IAC600, were obtained from a controlled field experiment performed in 2017 at the Dale Bumpers National Rice Research Center, Stuttgart, Arkansas. The variety Wells (PI 612439) is a long grain cultivar that has been grown on widespread acreage in Arkansas whereas IAC600 (PI 679960) is medium grain aromatic variety with purple/black bran that has been grown on limited acreage for specialty markets. The grain samples were produced using conventional management but using two irrigation treatments using previously published methods (Chen et al., 2021): season long flood (Flooded) and alternate wetting and drying (AWD), as well as two soil treatments: native soil and amended with MSMA (Yan et al., 2005). Prior to planting, 45 kg ha^−1^ phosphorous (P_2_O_5_) and 67 kg ha^−1^ potassium (K_2_O) were applied and incorporated. Just prior to planting, MSMA was applied to the soil at a rate of 5 kg ha^−1^. Plots were drill seeded on May 10, 2017 and then irrigated. 113 kg ha^−1^ of nitrogen as urea was applied approximately three weeks after emergence and the fields were then flooded. Weed and pest control followed standard cultural management procedures. On July 19, 2017 the AWD treatment was initiated and resulted in 3 dry down cycles completed by Sept. 7, 2017. Soil moisture was determined using a sensor (200SS Watermark, Irrometer, Riverside, CA) placed at 15-cm depth with data recorded hourly (900M, Irrometer, Riverside, CA). The target soil water potential was −20kP however due to rain events this was achieved only in the first AWD cycle followed by −15kP and -5kP in the two subsequent cycles, each followed by flooding. The grain was harvested at approximately 20 % moisture and then dried to 12 %. Rough rice was stored at 4 °C until bran processing. For these and other samples obtained in the USA, rice bran was produced, and heat stabilized as described in Zarei et al., 2018 prior to analysis.

### 2.4 Fermented rice bran

The rice bran RBT-300 (market sample II) was subjected to a 1-step and 2-step fermentation process as was previously described (Demissie et al., 2020), and is detailed here. The 1-step fermentation protocol involved the incubation of rice bran (RBT-300-2) for 24 h with strains: *Lactobacillus fermentum* ATCC 23271 (1LF), *L. paracasei* ATCC 21052_R1 (1LP)*, L. rhamnosus GG* (1LRGG), *Escherichia coli Nissle* 1917 (1ECN), and *Bifidobacterium longum* ATCC 55813 (1BL) and *Saccharomyces boulardii* (1SB). The commercial probiotic, Proboulardi (Metagenics Inc., San Clemente, CA), was used to isolate *S. boulardii*. Starter cultures were prepared 24–48 h in advance (at least 48 h for *Bifidobacterium*) by inoculating 500 μL of each probiotic (from 1 mL −80 °C freezer stock aliquots) into 500 mL of sterile deMann Rogasa Sharpe “MRS” or *Bifidobacterium* “BSM” broth. The following bacteria/media combinations were used: 500 mL sterile MRS +500 μL L*. fermentum*, 500 mL sterile MRS +500 μL L*. paracasei*, 500 mL sterile MRS +500 μL L*. rhamnosus* GG, 500 mL sterile MRS +500 μL E*. coli Nissle* 1917, 500 mL Sterile BSM +500 μL B*. longum*. *S. boulardii* fermentation was carried as previously described (Ryan et al., 2011). They were then incubated at 37 °C (in small microbial incubator). 1 kg of rice bran (RBT300-2) was mixed with each of the respective bacteria mentioned above. To estimate anaerobic fermentation conditions, mixtures were placed in an airtight stainless-steel container (fermentation chamber) and incubated at 37 °C. Approximately 48 h later, the slurry was then collected and frozen at −20 °C until lyophilization (Nealon et al., 2019).

The 2-step fermentation process involved incubation of rice bran (RBT-300-2) with yeast probiotic *S. boulardii* for 24 h followed by fermentation with each of the bacterial probiotics. These 2-step fermented rice brans are as listed: *S. boulardii* + *L. fermentum* (2LF), *S. boulardii* + *L. paracasei* (2LP)*, S. boulardii* + *L. rhamnosus GG* (2LRGG), *S. boulardii* + *E. coli Nissle* 1917 (2ECN), and *S. boulardii* + *B. longum* (2BL). To prepare the yeast starter culture and yeast fermentation, 1000 μL of *S. boulardii* was inoculated into 1000 mL sterile yeast nitrogen broth, amended with 0.5 % ammonium sulfate and 2 % dextrose, and incubated at 37 °C. 900 mL total of yeast + water was then added to 5 kg of rice bran (RBT-300-2). The yeast-fermented rice bran was separated into 5 equal 1-kg units. To each separated 1-kg unit of rice bran, 500 mL of probiotic (culture preparation described above) was added, so that each rice bran batch contained one of the five species. Each batch was then fermented in the environmental chamber for 48 h and then stored at −20 °C until lyophilization. Lyophilization utilized a Labconco Freezone 4.5 L Freeze Dry System affixed to Edwards RV5 vacuum pump (Marshall Scientific, Hampton, NH, USA).

### 2.5 Total As and As species quantification

Brooks Applied Labs (BAL) (Bothell, WA, USA) performed As speciation analysis (arsenite [As(III)], arsenate [As(V)], monomethylarsonic acid [MMAs], and dimethylarsinic acid [DMAs]), as well as total arsenic (As). BAL is accredited by the National Environmental Laboratory Accreditation Program (NELAP). iAs levels reported herein were calculated as the sum of the concentrations of As(III) and As(V) determined in each rice bran sample.

Arsenic species analysis was performed using method BAL-4101 Food by BAL-4116. Extraction was performed using Trifluoroacetic acid (TFA). Each extract was then assessed for As speciation via ion chromatography inductively coupled plasma collision reaction cell mass spectrometry (IC-ICP-CRC-MS), and then separated on an ion exchange column and quantified via inductively coupled plasma collision reaction cell mass spectrometry (ICP-CRC-MS).

Total arsenic (As) was prepared via a modified AOAC 2015.01 digestion. Resulting digests were analyzed for As using inductively coupled plasma triple quadrupole mass spectrometry (ICP-QQQ-MS). All sample results were method blank corrected and evaluated via reporting limits that accounted for aliquot size. All quality control results met acceptance criteria.

### 2.6 Quality assurance

Recovery calculations and the analytical quality was validated by the analysis of NIST (National Institute of Standards and Technology) standard reference materials (SRMs 1568b - rice flour and 1547 - peach leaves).

### 2.7 Statistics

Unpaired Student's t-tests were used to compare rice bran samples for total As, iAs, DMA, and MMA concentrations. The statistical tests were carried out using GraphPad Prism 8.0 software (GraphPad, USA). 95 % confidence intervals were assumed (p < 0.05). Descriptive statistics and linear regression to determine correlation between total As to iAs were performed using GraphPad Prism.

### 2.8 iAs exposure assessment

iAs risk exposure was calculated based on estimated daily intake (*EDI*), see Eq. (1) (Menon et al., 2020; Weber et al., 2019):

(1) $$EDI=\;\frac{C\times CR}{bw}$$

Where *EDI* (mg kg^−1^ bw^−1^ day^−1^) is the amount of iAs consumed; *C* (mg kg^−1^) is the concentration of iAs in rice bran; *CR* is (kg day^−1^) is the daily consumption rate of rice bran; *bw* (kg) is the average body weight. Mean body weights calculated were 9 kg for a 9.5 month old infant (CDC, 2000), 32 kg for a 10 year old child, and 80.7 kg for adults (Walpole et al., 2012). Consumption rates included for infants were 3 g of rice bran per day, 15 g per day for children and 30 g per day for adults.

*EDI* was then used to calculate the US EPA target hazard quotient (THQ). Reference oral dose (RfD) for iAs set by the US EPA (0.0003 mg kg^−1^ day^−1^) (EPA, 1991) was used for THQ calculations, see Eq. (2):

(2) $$THQ=\frac{EDI}{RfD}$$

A *THQ* quotient value less than one signifies no significant risk of non-carcinogenic effects.

To estimate the risk over time, lifetime cancer risk (*LCR*) was also calculated using *EDI*, and a slope factor (SF = 1.5 mg kg^−1^ day^−1^) as set by the US EPA, see Eq. (3):

(3) LCR=EDI×SF

The US EPA *LCR* acceptable upper limit is 1.0 × 10^−4^.

## 3 Results and discussion

### 3.1 Total As and As speciation

Total As, iAs, As(III), As(V), DMA, and MMA concentrations were quantified in 53 different rice bran samples (Table S1). Total As ranged from 0.017 to 1.86 mg kg^−1^ with a mean of 0.609 ± 0.393 mg kg^−1^. iAs (sum of As(III) and As(V)) ranged from 0.012 to 1.17 mg kg^−1^ with a mean of 0.463 ± 0.243 mg kg^−1^. Mean DMA concentration 0.077 ± 0.089 mg kg^−1^, range 0.007–0.352 mg kg^−1^. Mean MMA concentration was 0.017 ± 0.010 mg kg^−1^, range 0.007–0.039 mg kg^−1^. These values are in line with previously published studies on As speciation concentrations in brown rice and rice bran (Dai et al., 2014; Kim et al., 2018; Meharg et al., 2008; Ruangwises et al., 2012; Sun et al., 2008).

**Fig. 1:**
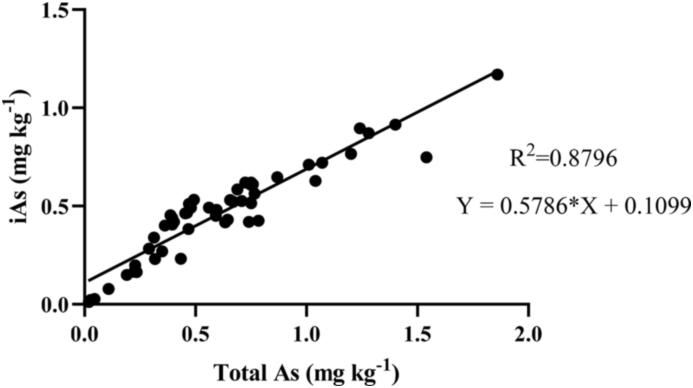
Linear regression illustrating the relationship between Total As (mg kg^−1^) and iAs (mg kg^−1^) for all rice bran samples (n = 53), R2 = 0.8796, p < 0.0001.

Linear regression modelling revealed the relationship between total grain As and iAs from all rice bran samples is shown in Fig. 1. The R^2^ value of 0.879 (p < 0.0001) indicated a strong linear relationship between total As and iAs in rice bran.

### 3.2 Geographic location impact on As concentration

Rice brans from 10 different countries were tested for As concentration and speciation. Countries included the US (n = 20), Cambodia (n = 6), India (n = 3), Egypt (n = 1), Guatemala (n = 2), Mali (n = 1), Madagascar (n = 2), Nepal (n = 1), Nicaragua (n = 1), and Thailand (n = 1). Note rice brans having atypical treatments (i.e. grown under MSMA (n = 4) soil conditions and fermented rice brans (n = 11)) were not included in this global assessment as to not skew the data (details of this are listed in Table S1).

**Fig. 2:**
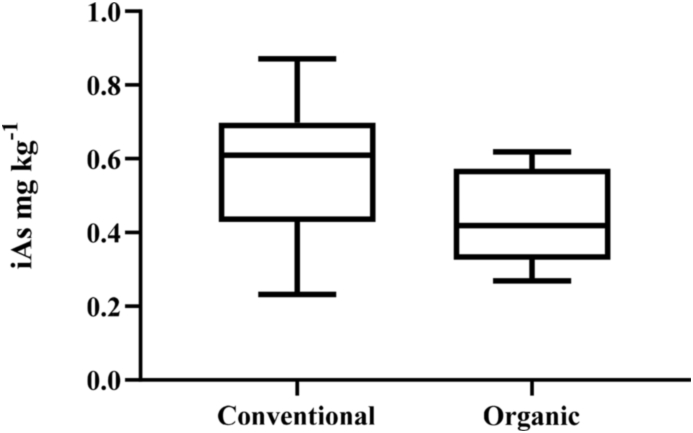
Conventional (n = 9) and organic (n = 5) iAs (mg kg^−1^) concentrations. Conventional and organic rice bran had mean iAs that were 0.565 ± 0.192 and 0.443 ± 0.135 mg kg^−1^, respectively. The solid line inside the box represents the median (0.610 and 0.419 mg kg^−1^) for conventional and organic rice bran respectively. No significant difference was found in conventional verses organic rice bran iAs concentrations (p = 0.235).

Mean total As was the highest in US rice bran (0.771 ± 0.310 mg kg^−1^), with 20 samples coming from very diverse growing areas in Texas, Arkansas, and California. The lowest As levels were in the rice bran from Guatemala (0.023 ± 0.006 mg kg^−1^). iAs concentrations for all countries is shown in Fig. 2. iAs was highest in the rice bran from Thailand (0.619 mg kg^−1^) followed by the samples from Cambodia (0.570 ± 0.042 mg kg^−1^) and from the USA (0.535 ± 0.177 mg kg^−1^). The lowest mean iAs was in the samples from Guatemala (0.017 ± 0.007 mg kg^−1^) and Madagascar (0.021 ± 0.009 mg kg^−1^). This demonstrates that As in rice bran can vary widely according to global source. iAs content in rice bran from Thailand was previously found to range from 0.599 to 0.673 mg kg^−1^ which aligns closely with our results where Thai rice bran had iAs concentration of 0.612 mg kg^−1^ (Ruangwises et al., 2012). In estimating As in global milled rice varieties, Zavala and Duxbury (2008) and Meharg et al. (2009) found the US rice varieties had some of the highest As contents, and interestingly, rice grown in India and Bangladesh had lower As concentrations, while Egypt had the lowest total As content. US rice bran in our results similarly had high As content while Indian and Egyptian rice bran samples had lower total and iAs content. There is no currently published data on rice or rice bran As content from Central America, which is likely due to its small contribution to global rice production. Based on the results in our study, Central American countries such as Guatemala and Nicaragua produced rice bran with low As content. However, further studies including a greater number of rice bran samples from this region should be carried out, as for example in Nicaragua, rice is primarily grown in one of four areas which are greatly affected by As contamination, including the Alluvial Aquifer of the Sebaco valley and neighbouring regions belonging to the Tertiary volcanic province, where a main alluvial aquifer is located (Delgado Quezada et al., 2020).

Other studies have found that As concentration can depend on harvesting time as well as with increasing milling ratios (Choi et al., 2014), factors also differ by region and country and their milling process. These major factors can account for differences observed between regions and cultivars.

### 3.3 Agronomic practice: irrigation and soil treatment

To examine irrigation technique and soil treatment impact on As concentration, bran was collected from rice grown using the AWD technique compared to rice grown under flooded conditions, and both in combination with native soil conditions and with MSMA applied.

**Fig. 3:**
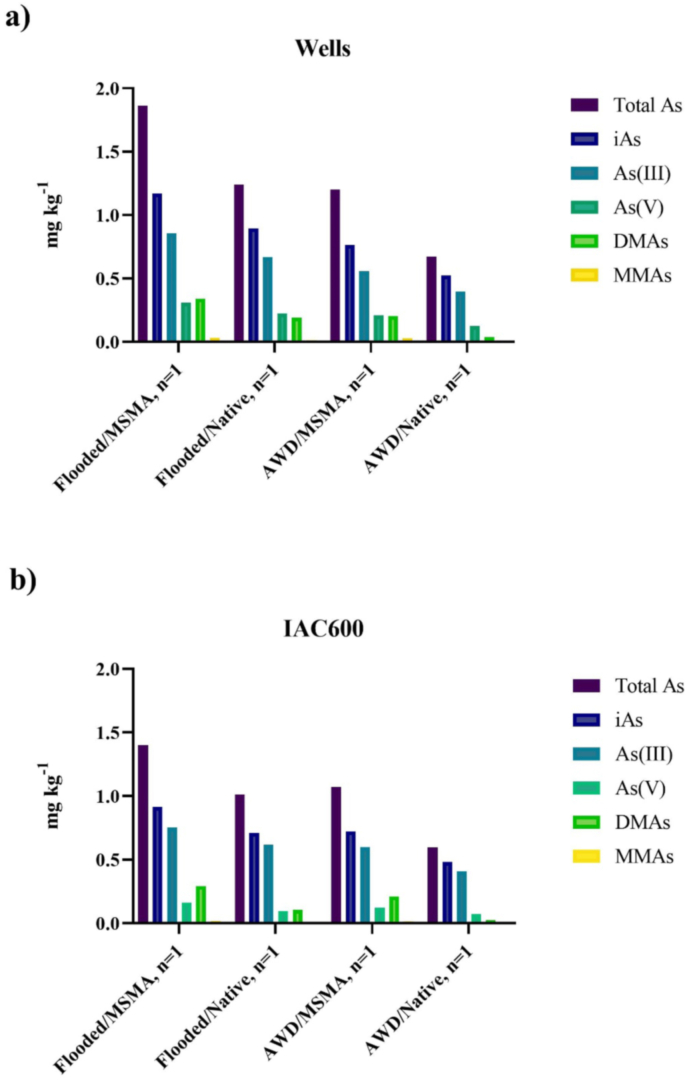
The concentration of As species (mg kg^−1^) for a) Wells rice bran and b) IAC600 rice bran. Wells and IAC600 varieties were each grown experimentally under the following conditions: Flooded/MSMA, Flooded/Native, AWD/MSMA, and AWD/Native.

Averaged over the two varieties grown on the native soil, the mean total As for AWD and flooded was 0.633 ± 0.054 and 1.12 ± 0.163 mg kg^−1^, respectively. iAs levels were 0.502 ± 0.029 mg kg^−1^ for AWD rice bran and 0.803 ± 0.130 mg kg^−1^ for flooded rice bran (Fig. 3). AWD rice bran also had lower mean DMA and MMA (0.031 ± 0.010 mg kg^−1^ and 0.003 ± 0.004 mg kg^−1^, respectively) compared to flooded rice bran DMA and MMA (0.149 ± 0.0622 mg kg^−1^ and 0.01 ± 0.006 mg kg^−1^, respectively). Although no significant difference was found between irrigation methods for total As, iAs, DMA, or MMA when tested on native soil, the AWD treatment had consistently lower As specie concentrations as has been previously reported (Carrijo et al., 2019; Norton et al., 2013; Xu et al., 2008).

Likewise, comparing bran from rice grown under the two irrigation techniques (AWD and flooded) on soils that had MSMA applied had mean total As concentrations of 1.14 ± 0.092 mg kg^−1^ and 1.63 ± 0.054 mg kg^−1^, for AWD and flooded, respectively. Mean iAs levels were 0.744 ± 0.032 mg kg^−1^ with AWD and 1.042 ± 0.181 mg kg^−1^ under flooded conditions. The same response to flooded and intermittent flood treatment on MSMA treated soils were also previously reported (Somenahally et al., 2011).

When comparing MSMA treated and native soil counterpart under AWD management, the MSMA treatment had significantly higher total As (*p* = 0.022), iAs (*p* = 0.016), and DMA (*p* = 0.002). Similarly, an evaluation of the two soil treatments under flooded conditions demonstrated that the MSMA treatment had higher total As, iAs, DMA, and MMA, though these concentrations were not significantly different from the native soil counterparts. However, DMA concentration in rice bran from flooded soil was significantly greater than that under AWD (*p* = 0.046).

Results from this study align with previous agronomic studies, where it is well established that As accumulation and speciation is largely influenced by water management practices, specifically that flooded fields have greater grain As concentrations as contrasted with AWD techniques (Arao et al., 2009; Ma et al., 2014; Norton et al., 2013; Wan et al., 2018; Xu et al., 2008; Yang et al., 2017). In a study by Norton et al. (2013) to investigate the effects of flooding conditions on rice grain As, they found that flooded rice consistently had higher accumulation of grain As in comparison to rice from non-flooded conditions. The anaerobic soil environment under flooded conditions is conducive for As uptake by plants. Under these flooded conditions, As is more readily released from the solid phase, becoming available for uptake by plants. Aerobic conditions are created when fields are left to desaturate before reflooding; these practices can also effectively reduce As accumulation (Orasen et al., 2019). AWD practices also benefit water conservation efforts and lessen greenhouse gas emissions in rice production (Linquist et al., 2015; Setyanto et al., 2017).

Although arsenical pesticides are not used in rice production, rice bran from MSMA treated soils were expected to have the highest As concentrations due to the direct addition of As to soil. The hazards surrounding use of arsenical compounds in agricultural soils has long been known, hence their current restricted use (Epps and Sturgis, 1940; Reed and Sturgis, 1936). As previously discussed, it has been found that flooded conditions provide conducive environments for As uptake, therefore it is understandable that rice bran from MSMA treated fields under flooded irrigation conditions have the highest As concentrations, as found by our study. Previous studies have also found that MSMA treated soils influenced As accumulation in rice (Farrow et al., 2015; Wauchope et al., 1982), which further supports our findings.

### 3.4 Agronomic practice: organic vs. conventional

**Fig. 4:**
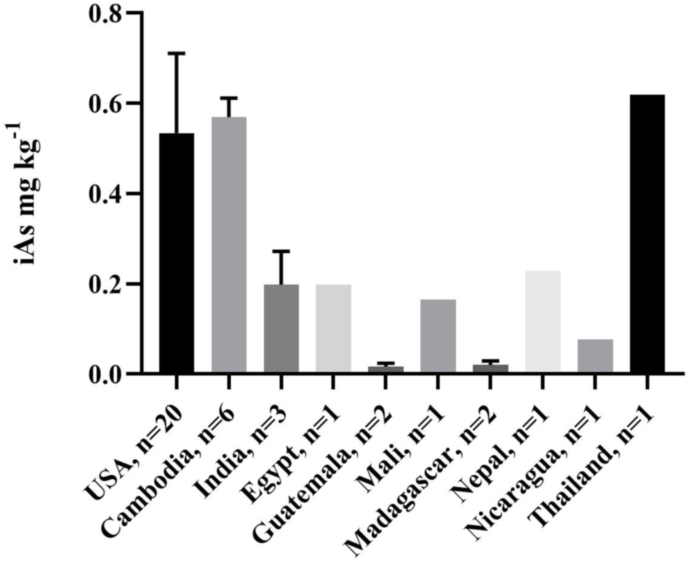
The iAs concentrations (mg kg^−1^) for rice bran collected across varieties from 10 different countries. The number of rice bran sources in each country is depicted by n.

Mean total As in conventionally (n = 28) and organically (n = 6) grown rice bran was 0.409 ± 0.267 and 0.755 ± 0.4416 mg kg^−1^, respectively. Fig. 4 depicts the mean iAs for conventional (0.374 ± 0.238 mg kg^−1^) and organic (0.494 ± 0.173 mg kg^−1^) rice bran. Mean DMA was 0.0443 ± 0.049 in conventional rice bran and was 0.123 ± 0.132 mg kg^−1^ in organic rice bran. Conventional and organic rice bran MMA concentrations were 0.008 ± 0.006 and 0.019 ± 0.012 mg kg^−1^, respectively. Thus, total As, iAs, DMA and MMA trended higher in organic rice bran as compared to conventional rice bran. Previous studies comparing conventional and organic grown brown rice have also found higher total As and iAs in organic rice as compared to conventional rice (Menon et al., 2020; Rahman et al., 2014; Segura et al., 2016). An important characteristic of organic farming is the enhancement of soil health through incorporation of vegetation and application of manures. The anaerobic environment of flooded rice paddies can increase As mobility and plant uptake to result in As accumulation in rice grains (Hossain et al., 2021; Ma et al., 2014; Norton et al., 2013). Market based surveys comparing conventional and organic grown brown rice have found higher total As and iAs in organic rice as compared to conventional rice (Menon et al., 2020; Rahman et al., 2014; Segura et al., 2016). However, others have reported no difference (Poletti et al., 2014). The multiple factors that impact As transformation and availability in the soil, differences in soil properties, cultural management practices, and choice of variety may explain these variable reports.

### 3.5 Rice bran fermentations

**Fig. 5:**
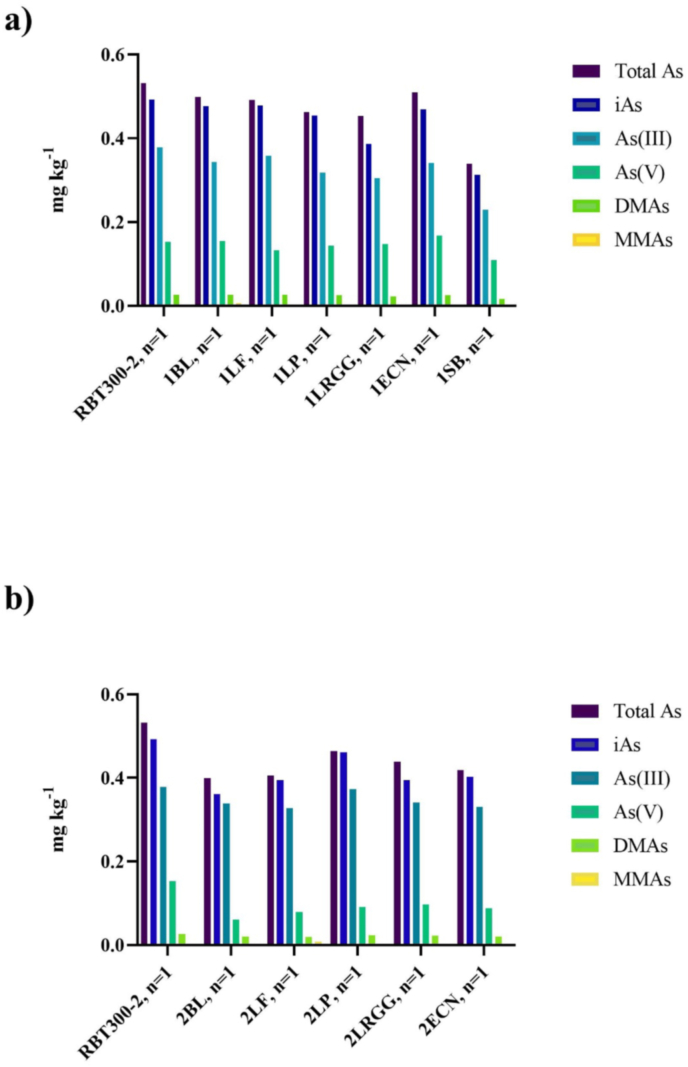
The impact of probiotic fermentation on rice bran As species (mg kg^−1^). No significant differences were detected between the RBT300-2 rice bran and a) 1-step fermentation and b) 2-step fermentation stages.

To better understand the effects of post-harvest treatment via fermentation on As levels in rice bran, a single rice bran (RBT-300-2) was fermented in a 1-step and 2-step fermentation process and tested for As levels (Fig. 5). There was a large decrease in all As species in step-1 fermented rice bran (1SB) verses RBT300-2, the original rice bran. There were not large differences in any As species in the other step-1 fermented rice brans as compared to RBT300-2. More of a decrease was found in step-2 fermented rice bran As species as compared to RBT300-2.

Though no significant difference was found in fermented 1-step or 2-step processes compared to the original rice bran (RBT300-2), fermentation may prove beneficial in prevention of As absorption after ingestion. This has been demonstrated in previous studies where bacteria such as *Lactobacillus rhamnosus* enriched yogurt reduced bioaccumulation of As in pregnant women and children (Bisanz et al., 2014). The addition of *Lactobacillus plantarum* has also shown the ability to chemically degrade pesticides in wheat (Dorđević et al., 2013). Lactobacilli and other commensal bacteria enhance gastrointestinal barrier function, reduce inflammation, and may have the potential to modulate host absorption and metabolism of xenobiotics (Meinl et al., 2009; Trinder et al., 2015). Further analysis of fermented rice brans therefore is warranted.

### 3.6 Rice bran As exposure and human health risk assessment

Concentrations of iAs in rice bran in our study aligned with prior studies, for example, Sun et al. (2008) observed that iAs in freshly milled rice bran from China and Bangladesh ranged from 1.17 to 3.61 mg kg^−1^ and 0.48–1.88 mg kg^−1^ in rice bran soluble products from the US and Japan. Similarly, in another study, rice bran collected from local markets in China varied in iAs from 0.159 to 1.295 mg kg^−1^ (Dai et al., 2014). With this information it is important to understand human health risk related with rice bran consumption.

The regulatory standard for iAs in infant foods is 0.1 mg kg^−1^ as set by the FDA (FDA, 2020). The concentrations of iAs in rice bran from this study ranged from 0.012 mg kg^−1^ to 1.12 mg kg^−1^. Heat-stabilized rice bran is considered a relatively new food ingredient for regular consumption in people. Given that some rice bran samples had iAs levels above 0.1 mg kg^−1^, it is essential to provide recommended ranges for daily consumption across the lifespan. Clinical studies by our team have tested relatively low daily doses of rice bran intake and with short durations (~1-6 months) for safety and tolerability as well as specific bioactive functions (e.g. blood lipid regulation, modulation of gut microbiota). Daily intake dosage recommendations are a major factor to consider for food safety regulations and dietary guidelines.

For EDI calculations, health risk was calculated according to two well established risk assessments: THQ and LCR (Table 1). Calculations were based on 3 scenarios: maximum (1.17 mg kg^−1^), median (0.465 mg kg^−1^), and minimum (0.012 mg kg^−1^) iAs rice bran concentrations instances. As expected, THQ and LCR exceeded regulation standards in Scenario 1 of the highest iAs rice bran where adults, infants, and children had a THQ value greater than 1 indicating there are possible adverse health effects and had LCR values less than 1.0 × 10^−4^. This exceeds the recommended LCR <1 × 10^−4^ to avoid carcinogenic risk. This rice bran used in Scenario 1 was grown under the MSMA/Flooded conditions and had purposely higher iAs levels and would likely be unrealistic out of these research settings.

**Table 1:** Health risk assessment of Target Hazard Quotient (THQ) and Lifetime Cancer Risk (LCR) calculations considering varying concentrations of iAs in rice bran for adults, children, and infants.

	Concentration iAs (mg kg^−1^)	Consumption Rate (g day^−1^)	BW (kg)	EDI (mg kg^−1^ day^−1^)	THQ (EDI RfD^−1^)	LCR (EDI^a^SF)
**Scenario 1** High Concentration						
Adult	1.17	30	80.7	4.35 × 10^−4^	1.45^a^	6.52 × 10^−4^^b^
Child	1.17	15	32	5.48 × 10^−4^	1.83^a^	8.23 × 10^−4^^b^
Infant	1.17	3	9	3.90 × 10^−4^	1.30^a^	5.85 × 10^−4^^b^
**Scenario 2** Median Concentration						
Adult	0.465	30	80.7	1.73 × 10^−4^	0.576	2.59 × 10^−4^^b^
Child	0.465	15	32	2.18 × 10^−4^	0.727	3.27 × 10^−4^^b^
Infant	0.465	3	9	1.55 × 10^−4^	0.517	2.33 × 10^−4^^b^
**Scenario 3** Low Concentration						
Adult	0.012	30	80.7	7.06 × 10^−6^	0.0235	1.06 × 10^−5^
Child	0.012	15	32	5.60 × 10^−6^	0.1880	8.44 × 10^−6^
Infant	0.012	3	9	4.00 × 10^−6^	0.0133	6.00 × 10^−6^

a THQ > 1.

b LCR > 1 × 10^−4^.

The median iAs in rice bran from all samples was used in the health risk Scenario 2 calculations to reduce the statistical impact of the two MSMA exposed rice bran outliers. When considering median iAs rice bran health risks, adult, children and infant THQ calculations did not exceed the threshold of 1 which indicates no adverse effects are expected. LCR calculations however did exceed 1 × 10^−4^ for adults, children, and infants.

In Scenario 3, the lowest iAs in rice bran THQ calculations did not surpass the recommended value of 1 in adults, children, or infants. LCR calculations also did not exceed 1.0 × 10^−4^ in any group. Both THQ and LCR are below the set recommendation limits, indicating no risk.

These calculations provide rationale for the importance in limiting As in rice bran, and the need to better understand what influences As concentrations in rice bran. Furthermore, while these dietary exposure assessment calculations provide insight into possible health risks, iAs bound rice bran may need additional differential, integrated assessments with respect to bioavailability, absorption, and the protective role of the microbiome in response to rice bran (Alava et al., 2013; Laparra et al., 2005; Nealon et al., 2019; Sheflin et al., 2017). A large body of literature has documented the toxicity of As in drinking water (Hopenhayn, 2006; Rahman et al., 2009; Villaescusa and Bollinger, 2008), however, As exposure from food sources, such as rice bran is more complex due to multiple essential nutrients and bioactive food components. In a previous study examining As absorption, it was found that the As bound to a rice matrix significantly lowered the bioaccessibility of As, and bioaccessibility also depended on the variety of rice (Alava et al., 2013). Importantly, in a recent study administering 1–5 g of rice bran daily to infants for 6-months, no significant difference was found between control and rice bran intervention groups in serum and stool As levels (Zambrana et al., 2021). In addition, in an *in vitro* analysis of the metabolism of As in rice bran on cultured human gut microbiota, it was discovered that the microbiota lowered As bioaccessibility and stimulated conversion of iAs to other As derivatives (Yin et al., 2019). These findings underscore the complexity in evaluating health risks associated with iAs-bound rice bran, and the need for techniques to lower As-rice bran exposure.

## 4 Study limitations

There are a few limitations to this study such as the lack of irrigation water and soil As assessments. Water and soil provide As to agroecological systems that influence rice-As levels (Dittmar et al., 2010). Furthermore, soil pH, organic matter, as well as biological and microbial soil conditions were not taken into account to influence As speciation and mobility in soil-plant systems (Abbas et al., 2018). Soil pH was also regarded as a major determinant in metal speciation, particularly under acidic soil conditions for higher As mobility and phyto-availability (Signes-Pastor et al., 2007). Future studies will need to assess physico-chemical characteristics of the environment when using As speciation, mobility, and bioavailability to limit As uptake in rice plants (Khalid et al., 2017; Singh and Srivastava, 2020).

## 5 Conclusions

This quantitative investigation of As speciation in rice brans with global relevance to food safety and health was conducted for an emerging human food ingredient that will be consumed across the lifespan and from diverse environments, agricultural and nutritional settings. Multiple factors contributed to the range in rice bran As species concentrations, with agronomic practices and cultivars as major influencers for As uptake into the grain. Adopting AWD practices may help reduce As accumulation where dosage levels exceed thresholds of relevance to human health risk. Future studies aimed at limiting As in rice bran through agronomic techniques such as irrigation practices are warranted. Postharvest techniques to lower As in rice bran should also be explored with respect to identifying microbes that ferment rice bran in a manner that can support As metabolism into harmless derivatives and less toxic forms. Comparisons for As levels in rice bran over yearly growing seasons and from climate change merits attention by geographic location given that As in the soil may fluctuate and water availability impacted, and thus changes to uptake by rice plant varieties may further influence levels in rice bran. Given the suite of health benefits and increasing attention to rice bran as a superfood, it is imperative to examine agronomic and postharvest influencers of As species concentrations and seek efforts to reduce As exposure from rice bran consumption.

## Author statement

The authors' responsibilities were as follows – EPR: designed research and maintained study oversight; AMW BAB and EPR contributed to study design; BAB, AM, MML, SBD, SV, OK, FW, and EPR: conducted research and sample collection; AMW conducted data analysis; AMW, AM, MML, SBD and SV, FW, and EPR wrote and edited the paper; EPR had primary responsibility for the final product. All authors read and approved the final manuscript. The authors declare that they have no conflicts of interest with the publication of this work.

## Declaration of competing interest

The authors declare that they have no known competing financial interests or personal relationships that could have appeared to influence the work reported in this paper.
