# Supplementary material for: Arsenic speciation in rice bran: Agronomic practices, postharvest fermentation, and human health risk assessment across the lifespan
Source: Environ Pollut. 2021 Dec 1;290:117962. doi: 10.1016/j.envpol.2021.117962 (PMC8556161; doi:10.1016/j.envpol.2021.117962)
Supplement: Multimedia component 1 [file mmc1.docx]

| **Supplemental Table 1. Additional rice bran details and arsenic speciation concentrations. (* If name of variety from a country is unknown)** | | | | | | | | | | |
| --- | --- | --- | --- | --- | --- | --- | --- | --- | --- | --- |
| **Sample** | **Rice Bran Variety** | **Growing Condition/Treatment** | **Rice Bran Color** | **Origin** | **Total As (mg/kg)** | **iAs (mg/kg)** | **As(III) (mg/kg)** | **As(V) (mg/kg)** | **DMAs (mg/kg)** | **MMAs (mg/kg)** |
| 1 | Jazzman | Organic | Brown | Brinkley, AR, USA | 0.349 | 0.269 | 0.233 | 0.037 | 0.045 | 0.009 |
| 2 | Jazzman | Conventional | Brown | Brinkley, AR, USA | 0.56 | 0.492 | 0.402 | 0.090 | 0.053 | 0.007 |
| 3 | Carolina Gold | Organic | Brown | Beaumont, TX, USA | 0.741 | 0.419 | 0.328 | 0.090 | 0.220 | 0.039 |
| 4 | Carolina Gold | Conventional | Brown | Beaumont, TX, USA | 0.784 | 0.426 | 0.335 | 0.091 | 0.225 | 0.039 |
| 5 | Della 2 | Organic | Brown | Brinkley, AR, USA | 0.468 | 0.383 | 0.281 | 0.103 | 0.033 | 0.021 |
| 6 | Della 2 | Conventional | Brown | Brinkley, AR, USA | 0.758 | 0.610 | 0.483 | 0.127 | 0.076 | 0.013 |
| 7 | Wells | Organic | Brown | Brinkley, AR, USA | 0.708 | 0.526 | 0.429 | 0.097 | 0.052 | 0.011 |
| 8 | IAC600 | Conventional | Purple | Stuttgart, AR, USA | 0.434 | 0.232 | 0.202 | 0.030 | 0.051 | ≤ 0.005 |
| 9 | IAC600 | Conventional | Purple | Beaumont, TX, USA | 0.868 | 0.647 | 0.543 | 0.103 | 0.111 | 0.008 |
| 10 | IAC600 | Flooded-MSMA | Purple | Stuttgart, AR, USA | 1.4 | 0.914 | 0.753 | 0.160 | 0.292 | 0.018 |
| 11 | IAC600 | AWD-Native | Purple | Stuttgart, AR, USA | 0.595 | 0.481 | 0.410 | 0.071 | 0.024 | ≤ 0.006 |
| 12 | IAC600 | Flooded-Native | Purple | Stuttgart, AR, USA | 1.01 | 0.711 | 0.617 | 0.094 | 0.105 | ≤ 0.006 |
| 13 | IAC600 | AWD-MSMA | Purple | Stuttgart, AR, USA | 1.07 | 0.721 | 0.598 | 0.123 | 0.210 | 0.015 |
| 14 | Wells | Conventional | Brown | Beaumont, TX, USA | 0.645 | 0.431 | 0.349 | 0.081 | 0.034 | ≤ 0.006 |
| 15 | Wells | Conventional | Brown | Stuttgart, AR, USA | 1.28 | 0.871 | 0.712 | 0.158 | 0.139 | 0.013 |
| 16 | Wells | Flooded-MSMA | Brown | Stuttgart, AR, USA | 1.86 | 1.17 | 0.858 | 0.309 | 0.340 | 0.032 |
| 17 | Wells | AWD-Native | Brown | Stuttgart, AR, USA | 0.671 | 0.522 | 0.397 | 0.125 | 0.037 | ≤ 0.006 |
| 18 | Wells | Flooded-Native | Brown | Stuttgart, AR, USA | 1.24 | 0.895 | 0.669 | 0.226 | 0.193 | 0.014 |
| 19 | Wells | AWD-MSMA | Brown | Stuttgart, AR, USA | 1.2 | 0.766 | 0.557 | 0.209 | 0.202 | 0.027 |
| 20 | Calrose | Conventional | Brown | Nelson, CA, USA | 0.592 | 0.451 | 0.323 | 0.127 | 0.035 | 0.007 |
| 21 | Tesanai 2 | Organic | Brown | Beaumont, TX, USA | 1.54 | 0.748 | 0.615 | 0.133 | 0.352 | 0.024 |
| 22 | Tesanai 2 | Conventional | Brown | Stuttgart, AR, USA | 1.04 | 0.628 | 0.521 | 0.107 | 0.092 | 0.010 |
| 23 | Guatemala*, Mill I | Conventional | Brown | Guatemala | 0.019 | 0.012 | ≤ 0.004 | 0.010 | ≤ 0.006 | ≤ 0.006 |
| 24 | Guatemala*, Mill II | Conventional | Brown | Guatemala | 0.0271 | 0.022 | 0.006 | 0.016 | ≤ 0.006 | ≤ 0.006 |
| 25 | Nicaragua* | Conventional | Brown | Nicaragua | 0.108 | 0.078 | 0.051 | 0.027 | ≤ 0.005 | ≤ 0.005 |
| 26 | Madagascar* | Lowland, Conventional | Red | Ampitatafira village, Madagascar | 0.0452 | 0.027 | 0.018 | 0.010 | ≤ 0.006 | ≤ 0.006 |
| 27 | Madagascar* | Highland, Conventional | Red | CHD, Madagascar | 0.0171 | 0.014 | 0.010 | ≤ 0.004 | ≤ 0.006 | ≤ 0.006 |
| 28 | Jasmine, Mill I | Wet Season, Conventional | Brown | Cambodia | 0.656 | 0.531 | 0.332 | 0.199 | 0.038 | ≤ 0.006 |
| 29 | Jasmine, Mill II | Wet Season, Conventional | Brown | Cambodia | 0.751 | 0.514 | 0.333 | 0.180 | 0.038 | ≤ 0.005 |
| 30 | Jasmine, Mill I | Dry Season, Conventional | Brown | Cambodia | 0.688 | 0.585 | 0.488 | 0.097 | 0.054 | ≤ 0.005 |
| 31 | Jasmine, Mill II | Dry Season, Conventional | Brown | Cambodia | 0.766 | 0.563 | 0.466 | 0.097 | 0.052 | ≤ 0.006 |
| 32 | Non-Jasmine, Mill I | Conventional | Brown | Cambodia | 0.749 | 0.608 | 0.365 | 0.243 | 0.047 | ≤ 0.006 |
| 33 | Non-Jasmine, Mill II | Conventional | Brown | Cambodia | 0.751 | 0.619 | 0.388 | 0.231 | 0.046 | ≤ 0.006 |
| 34 | New Dehli, India* | Conventional | Brown | India | 0.29 | 0.283 | 0.213 | 0.070 | 0.010 | ≤ 0.005 |
| 35 | Egypt* | Conventional | Brown | Egypt | 0.228 | 0.198 | 0.129 | 0.069 | 0.007 | ≤ 0.005 |
| 36 | Chennula | Conventional | Red | India | 0.235 | 0.164 | 0.099 | 0.065 | 0.015 | ≤ 0.006 |
| 37 | Njavara | Conventional | Red | India | 0.19 | 0.149 | 0.102 | 0.047 | ≤ 0.006 | ≤ 0.006 |
| 38 | Nepal* | Conventional | Brown | Jeera Mosino, Nepal | 0.317 | 0.230 | 0.116 | 0.114 | 0.010 | ≤ 0.006 |
| 39 | Urmatt | Organic | Brown | Thailand | 0.724 | 0.619 | 0.432 | 0.187 | 0.040 | 0.008 |
| 40 | Rice Bran Technology 300, market sample I (RBT300-1) | Conventional | Brown | California, USA | 0.634 | 0.417 | 0.283 | 0.134 | 0.029 | ≤ 0.005 |
| 41 | Khao-gaew | Conventional | Brown | Mali | 0.224 | 0.165 | 0.101 | 0.064 | 0.016 | ≤ 0.006 |
| 42 | Rice Bran Technology 300, market sample II (RBT300-2) | Conventional | Brown | California, USA | 0.532 | 0.493 | 0.379 | 0.153 | 0.027 | ≤ 0.005 |
| 43 | 1-step Bifidobacterlum longum (1BL) | Fermented (RBT300-2) | Brown | Fort Collins, CO, USA | 0.499 | 0.477 | 0.344 | 0.155 | 0.027 | 0.007 |
| 44 | 1-step S.boulardi (1SB) | Fermented (RBT300-2) | Brown | Fort Collins, CO, USA | 0.339 | 0.313 | 0.230 | 0.109 | 0.017 | ≤ 0.006 |
| 45 | 1-step L. Ferentum (1LF) | Fermented (RBT300-2) | Brown | Fort Collins, CO, USA | 0.492 | 0.479 | 0.359 | 0.133 | 0.027 | ≤ 0.006 |
| 46 | 1-step L. paracase (1LP) | Fermented (RBT300-2) | Brown | Fort Collins, CO, USA | 0.463 | 0.454 | 0.318 | 0.144 | 0.026 | ≤ 0.006 |
| 47 | 1-step L. rhamnosus GG (1LRGG) | Fermented (RBT300-2) | Brown | Fort Collins, CO, USA | 0.453 | 0.387 | 0.305 | 0.148 | 0.023 | ≤ 0.005 |
| 48 | 1-step E. coli Nissle (1ECN) | Fermented (RBT300-2) | Brown | Fort Collins, CO, USA | 0.510 | 0.47 | 0.342 | 0.168 | 0.026 | ≤ 0.006 |
| 49 | 2-step B. Longum (2BL) | Fermented (RBT300-2) | Brown | Fort Collins, CO, USA | 0.400 | 0.362 | 0.339 | 0.061 | 0.021 | ≤ 0.005 |
| 50 | 2-step E. Coli Nissle (2ECN) | Fermented (RBT300-2) | Brown | Fort Collins, CO, USA | 0.419 | 0.403 | 0.331 | 0.088 | 0.021 | ≤ 0.005 |
| 51 | 2-step L Paracase (2LP) | Fermented (RBT300-2) | Brown | Fort Collins, CO, USA | 0.465 | 0.462 | 0.374 | 0.091 | 0.024 | ≤ 0.005 |
| 52 | 2-step L. Fermentum (2LF) | Fermented (RBT300-2) | Brown | Fort Collins, CO, USA | 0.406 | 0.395 | 0.328 | 0.079 | 0.020 | 0.009 |
| 53 | 2-step L. Rhamnosus GG (2LRGG) | Fermented (RBT300-2) | Brown | Fort Collins, CO, USA | 0.439 | 0.395 | 0.341 | 0.098 | 0.023 | ≤ 0.006 |
